# Radiologic 3D tumor volume predicts cervical metastasis in oral squamous cell carcinoma

**DOI:** 10.1007/s00784-026-07043-y

**Published:** 2026-07-23

**Authors:** A. Schmitz, V. Corneo, C. Rendenbach, C. Doll, F. Elsholtz, K. Kreutzer, F. Mrosk, M. Alfertshofer, M. Heiland, S. Koerdt

**Affiliations:** 1 Department of Oral and Maxillofacial Surgery, Charité – Universitätsmedizin Berlin, corporate member of Freie Universität Berlin and Humboldt-Universität Zu Berlin, Augustenburger Platz 1, 13353 Berlin, Germany; 2 Department of Radiology, Charité - Universitätsmedizin Berlin, corporate member of Freie Universität Berlin, Humboldt-Universität Zu Berlin, Charitéplatz 1, 10117 Berlin, Germany

**Keywords:** OSCC, Volumetry, Tomography, Metastasis, Staging, Biomarkers

## Abstract

**Objectives:**

To test whether pre-operative primary CT-based 3D tumor volume (TV) improves prediction of cervical spread in oral squamous cell carcinoma (OSCC) beyond conventional size metrics.

**Materials and methods:**

We retrospectively analyzed 132 consecutive primary OSCC patients (2014–2019). Contrast-enhanced CT datasets were semi-automatically segmented to obtain 3D TV (cm^3^), verified by a second observer. Multivariable logistic regression related log-TV to pathologic lymph-node metastasis (LNM), extranodal extension (ENE), and skip metastasis. Performance of radiologic TV, depth of invasion (DOI), and ellipsoid pathologic volume was compared by ROC/AUC and decision-curve analyses.

**Results:**

Median CT-derived TV was 12.6 cm^3^. Log-TV independently predicted LNM (odds ratio 3.00, 95% CI 1.21–7.68; AUC 0.815), outperforming DOI (AUC 0.541) and pathologic volume (AUC 0.498). A non-linear pattern linked very small (< 1.8 cm^3^) and large (> 16 cm^3^) tumors to increased skip-metastasis probability (AUC 0.733). Radiologic and pathologic volumes showed minimal concordance (R^2^ = 0.0002). Decision-curve analysis demonstrated consistent net benefit of the volume model across clinically relevant threshold probabilities for elective neck dissection.

**Conclusions:**

CT-based 3D volumetry is an independent pre-operative predictor of cervical LNM, flags tumors prone to skip spread, and offers greater clinical utility than linear measures.

**Clinical relevance:**

Integrating radiologic volumetry into staging could refine nodal management—particularly in clinically node-negative patients—by supporting risk-adapted selection between sentinel lymph-node biopsy and elective neck dissection; prospective validation is warranted.

**Supplementary Information:**

The online version contains supplementary material available at 10.1007/s00784-026-07043-y.

## Introduction

Oral squamous cell carcinoma (OSCC) constitutes roughly 90% of oral malignancies and remains a major contributor to the global cancer burden [1, 2]. Despite improved surgery, multimodal therapy, and earlier detection, 5-year survival has stalled at 64%−66%, and disease-free survival (DFS) reaches up to 80 months, depending on tumor- and patient-specific factors [3, 4].

Accurate staging is critical for individualized treatment planning. Cervical lymph node metastasis (LNM) is a central prognostic determinant that shapes survival and guides surgical as well as radiotherapeutic decisions [3, 4]. Preoperative identification of LNM remains challenging. Occult LNM occur in up to 42% of OSCC cases [5, 6]. CT and MRI evaluate nodal size and morphology, yet their sensitivity and specificity are only moderate (72% to 81%), leading to under- and overtreatment. Reporting systems like Node-RADS aim to standardize nodal assessment, but still await validation in large patient cohorts. PET-CT offers higher accuracy and a strong negative predictive value, yet routine use is limited by cost, access and reduced sensitivity for microscopic disease in cN0 patients. Consequently, elective neck dissection remains the standard of care in early-stage OSCC [7–10]. To overcome these limitations, models that integrate imaging and clinicopathologic variables have been proposed. Although nomograms and machine-learning approaches are promising, they still rely on two-dimensional metrics, maximum diameter and depth of invasion (DOI), which inadequately capture total tumor burden [10, 11, 13–15].

Recent imaging advances enable three-dimensional (3D) tumor volumetry, providing a more comprehensive and biologically relevant measure of tumor mass. Unlike linear metrics, volumetry captures tumor spatial complexity and may correlate more closely with nodal spread [11]. Earlier studies associate 3D tumor volume with nodal involvement and poorer survival in head and neck cancers [13, 14]. Nonetheless, its predictive value in OSCC, particularly in clinically node-negative (cN0) disease, remains incompletely defined.

We therefore evaluate whether preoperative 3D radiologic tumor volume (rTV) predicts LNM, extranodal extension (ENE), and skip metastases in OSCC. We further compare rTV and pathologic volumes (pTV), benchmark its performance against DOI, and explore prognostic utility through survival decision-curve analysis to inform individualized staging and management.

## Methods

### Study design

Inclusion criteria comprised adult patients with biopsy-proven primary OSCC who subsequently underwent primary surgical resection and had available preoperative contrast-enhanced CT imaging.

Exclusion criteria included prior head and neck malignancy, distant metastases at diagnosis, incomplete clinical data, non-contrast CT scans, or CT datasets with relevant motion or metal artifacts precluding reliable volumetric segmentation.

### Ethics statement

The study was approved by the institutional ethics committee of Charité – Universitätsmedizin Berlin (EA1/249/24) and was conducted in accordance with the ethical standards outlined in the Declaration of Helsinki.

### Clinical data collection

Baseline demographics, risk factors, TNM stage, histologic grade, treatment details, and nodal status (including ENE and skip metastasis) were abstracted from electronic records by blinded reviewers. Skip metastasis was defined as cervical lymph node metastasis occurring in non-adjacent nodal levels without involvement of the expected first-echelon lymph nodes, with cervical lymph node levels classified according to the Robbins neck level classification [12].

### Volumetric assessment

**Fig. 1:**
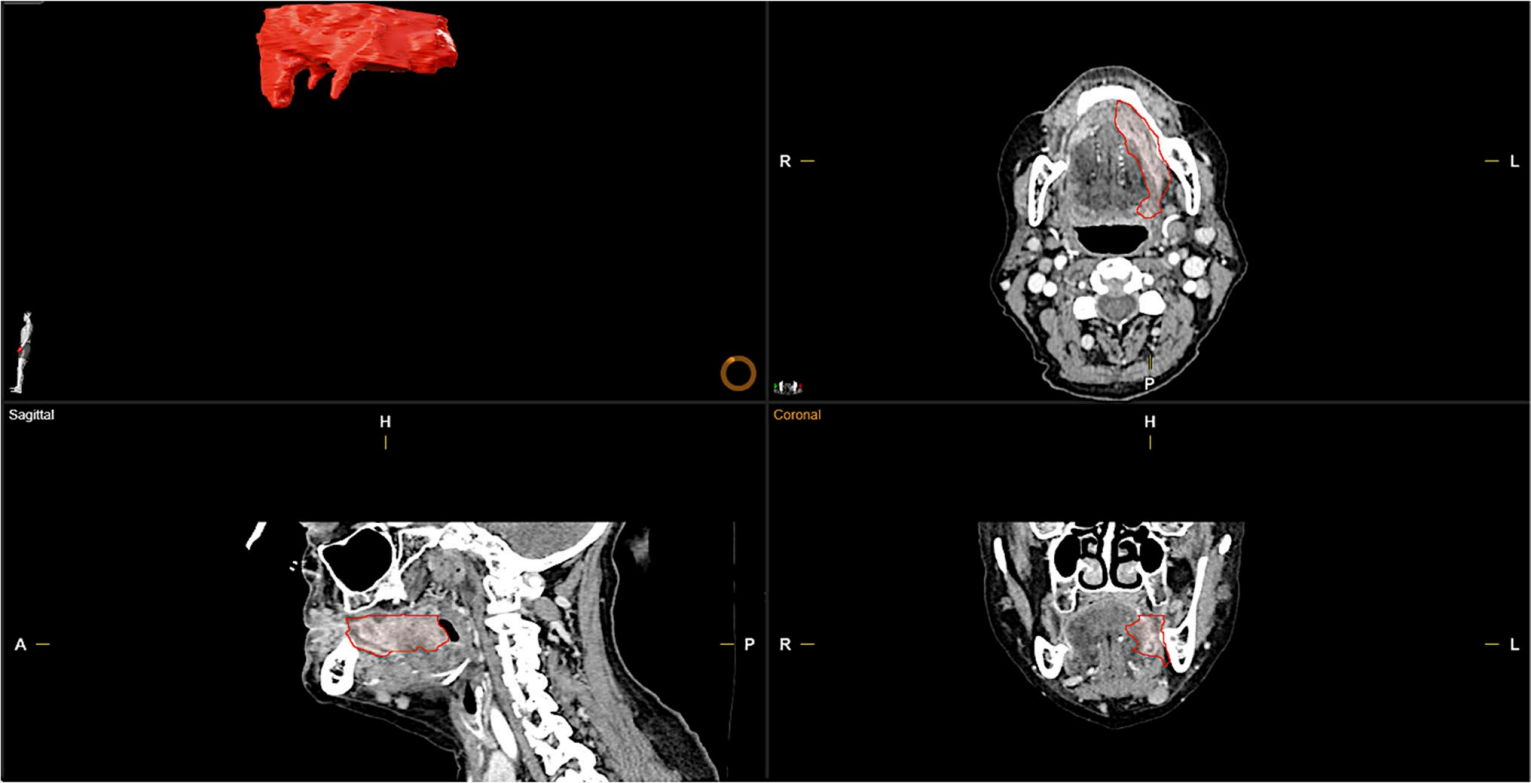
Three-Dimensional Segmentation of OSCC on Contrast-Enhanced CT. Manual contours (red) were applied on axial and sagittal views; the segmentation software interpolated intervening slices to generate the 3-D rendering (inset), yielding a tumor volume of 17.8 cm^3^. CT indicates computed tomography. The primary segmentation was performed by a resident in oral and maxillofacial surgery who had been trained in volumetric segmentation protocols. All segmentations were independently reviewed and, if necessary, corrected by an experienced consultant surgeon (> 10 years of head and neck oncologic experience). Both raters were blinded to nodal status, survival data and histopathological results at the time of segmentation

Preoperative rTV was defined as the contrast-enhancing volume of the primary tumor on CT. CT imaging was performed using contrast-enhanced multidetector CT scanners with reconstructed slice thicknesses in the range of 1.0–3.0 mm, which is suitable for three-dimensional volumetric segmentation. 3D tumor segmentation was performed using Brainlab Elements software (Brainlab AG, Munich, Germany, version 3.1.42) and the SmartBrush® tool following a standardized semi-automated workflow (Fig. 1).

Tumor margins were manually delineated on selected axial slices and orthogonal planes, typically sagittal, enabling interpolation and 3D reconstruction. rTV was automatically calculated in cm^3^ based on the segmented contrast-enhancing tumor area. All segmentations were independently reviewed and, if necessary, corrected by a second experienced investigator using the same software environment. Scans with motion artifacts or insufficient contrast enhancement were excluded. Segmenters were blinded to nodal status, survival outcomes, and histopathological results.

Primary pTV was not directly measured as a three-dimensional entity. Instead, pTV was estimated from routinely reported histopathological parameters of the resection specimen, including maximum tumor diameter (D_max), orthogonal diameter (D_orth), and DOI. pTV was calculated using an ellipsoid approximation formula:

$$TV=\pi /6\times D\_max\times D\_orth\times DOI$$

### Statistical analysis

Statistical analyses were performed using R (version 4.4). Continuous variables are reported as mean ± standard deviation or median with interquartile range (IQR), as appropriate; categorical variables are presented as counts and percentages. rTV was log-transformed prior to analysis due to right-skewed distribution.

The primary endpoint was pathologic LNM. Secondary endpoints included ENE, skip metastasis, overall survival (OS), and DFS. Associations between clinical and imaging variables and binary endpoints were assessed using logistic regression. Multivariable models included rTV and predefined clinicopathologic covariates (age, tumor subsite, histologic grade, and DOI). Model fit was assessed using the Akaike information criterion (AIC), and calibration was evaluated using the Hosmer–Lemeshow goodness-of-fit test.

Model discrimination was quantified using receiver-operating-characteristic (ROC) curves and the area under the curve (AUC) with 95% confidence intervals. Comparisons between AUCs were performed using DeLong’s test for correlated ROC curves. Decision-curve analysis was applied to evaluate the net clinical benefit of volume-based models across clinically relevant threshold probabilities.

Survival outcomes were analyzed as exploratory endpoints using Kaplan–Meier estimates with log-rank testing and Cox proportional hazards regression. All statistical tests were two-sided, and a P value < 0.05 was considered statistically significant.

## Results

### Patient characteristic

A total of 132 patients with primary OSCC were included. Baseline demographic, tumor-related, and treatment characteristics are summarized in Table 1. The majority of tumors were located in the tongue or floor of the mouth, and neck dissection was performed in 83.5% of patients.

**Table 1:** Baseline patient, tumor, and treatment characteristics

Characteristic	Overall cohort (n = 132)	cN0 patients (n = 78)	cN0 + ND (n = 46)	ND patients (n = 106)
Age, years (mean ± SD)	63.7 ± 13.8	63.1 ± 13.4	62.8 ± 13.1	64.2 ± 14.0
Male sex, n (%)	78 (59.1)	44 (56.4)	26 (56.5)	62 (58.5)
Primary tumor subsite, n (%)				
Tongue	64 (48.5)	36 (46.2)	21 (45.7)	52 (49.1)
Floor of mouth	59 (45.0)	37 (47.4)	22 (47.8)	48 (45.3)
Other subsites†	9 (6.8)	5 (6.4)	3 (6.5)	6 (5.7)
Neck dissection performed, n (%)	106 (83.5)	46 (59.0)	46 (100)	106 (100)
Type of neck dissection, n (%)	–	–	–	
Selective ND	–	–	–	58 (54.7)
Modified radical ND	–	–	–	16 (15.1)
Radical ND	–	–	–	32 (30.2)
Pathologic nodal status, n (%)	–	–		
pN0	–	–	20 (43.5)	38 (35.8)
pN +	–	–	26 (56.5)	68 (64.2)
Extranodal extension (ENE), n (%)	–	–	–	24 (22.6)
Skip metastasis, n (%)	–	–	–	20 (18.9)
Radiologic primary tumor volume, cm^3^ (median)	12.6	10.9	11.2	13.4

^†^Other subsites include upper and lower gingiva and buccal mucosa

Percentages refer to the respective column denominator. Pathologic nodal status, extranodal extension, and skip metastasis were assessed only in patients undergoing neck dissection

Abbreviations: cN, clinical nodal status; ND, neck dissection; pN, pathologic nodal status

### Radiologic tumor volume predicts lymph node metastasis

In multivariable logistic regression, log-transformed rTV was a significant independent predictor of pathologic LNM (OR = 3.00; 95% CI, 1.21–7.68), whereas DOI, histologic grade, tumor subsite, and age were not significantly associated with LNM (Supplementary Table 1). Overall model fit was acceptable (AIC = 101.01).

**Fig. 2:**
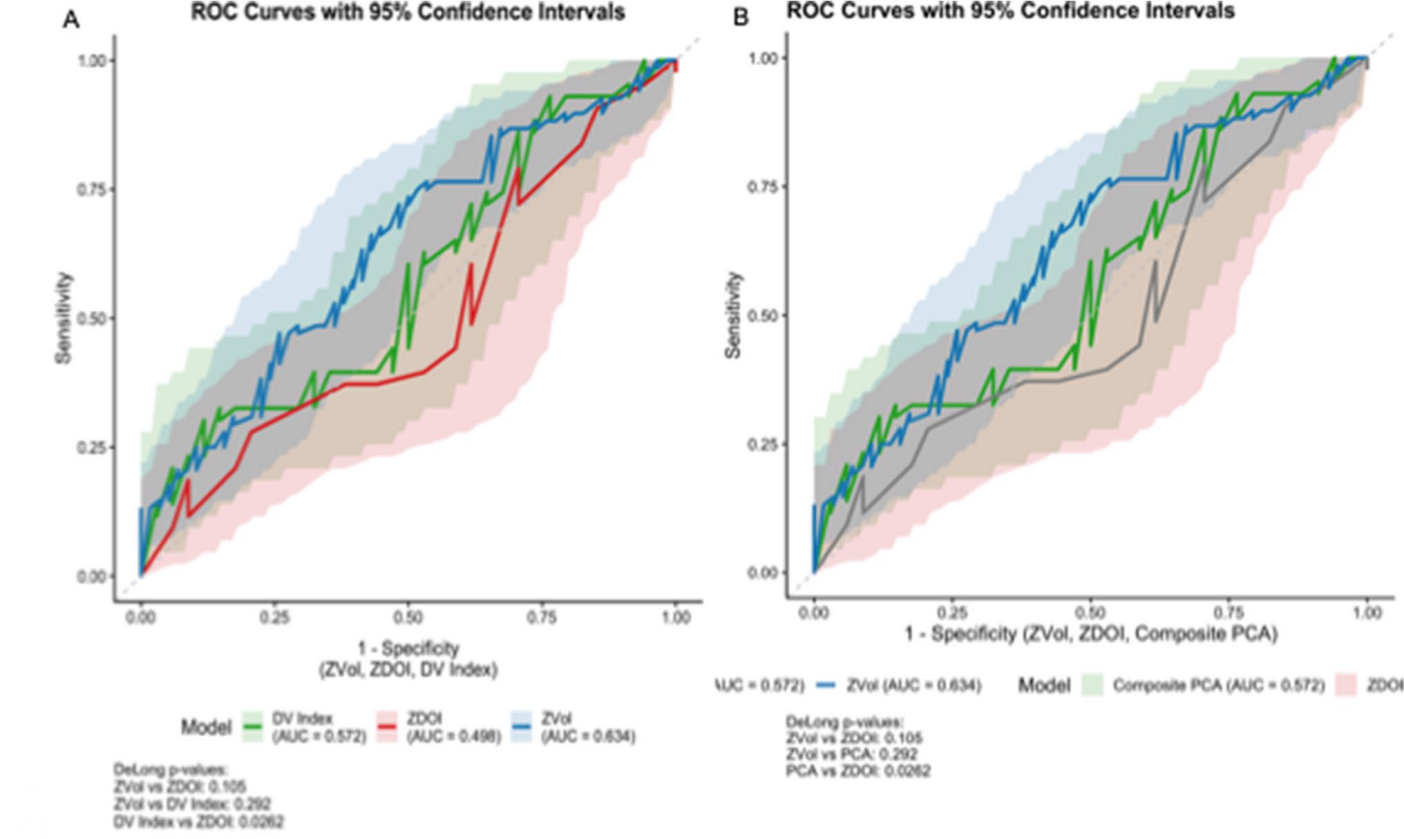
Receiver-Operating-Characteristic (ROC) Curves for Predicting Pathologic Lymph-Node Metastasis. A, ROC curves (with 95% CIs) for standardized tumor volume (ZVol), standardized depth of invasion (ZDOI), and the combined DV Index. ZVol yielded the highest discrimination (AUC 0.63); the DV Index improved on ZDOI (P = 0.03, DeLong test) but not on ZVol (P = 0.29). B, A principal-component (PCA) score (AUC 0.57) mirrored the DV Index, again outperforming ZDOI but not ZVol. AUC indicates area under the curve; CI, confidence interval; DOI, depth of invasion; DV, depth–volume; LNM, lymph-node metastasis; PCA, principal-component analysis; ROC, receiver-operating characteristic

ROC analysis demonstrated superior discriminatory performance of rTV for predicting LNM (AUC = 0.634) compared with depth of invasion (AUC = 0.498) and the combined depth–volume (DV) index (AUC = 0.572). Comparisons of AUCs confirmed that rTV significantly outperformed DOI (*P* = 0.026, DeLong test), while composite models did not improve discrimination beyond rTV alone (Fig. 2A–B).

### Decision-curve analysis for LNM and ENE

**Fig. 3:**
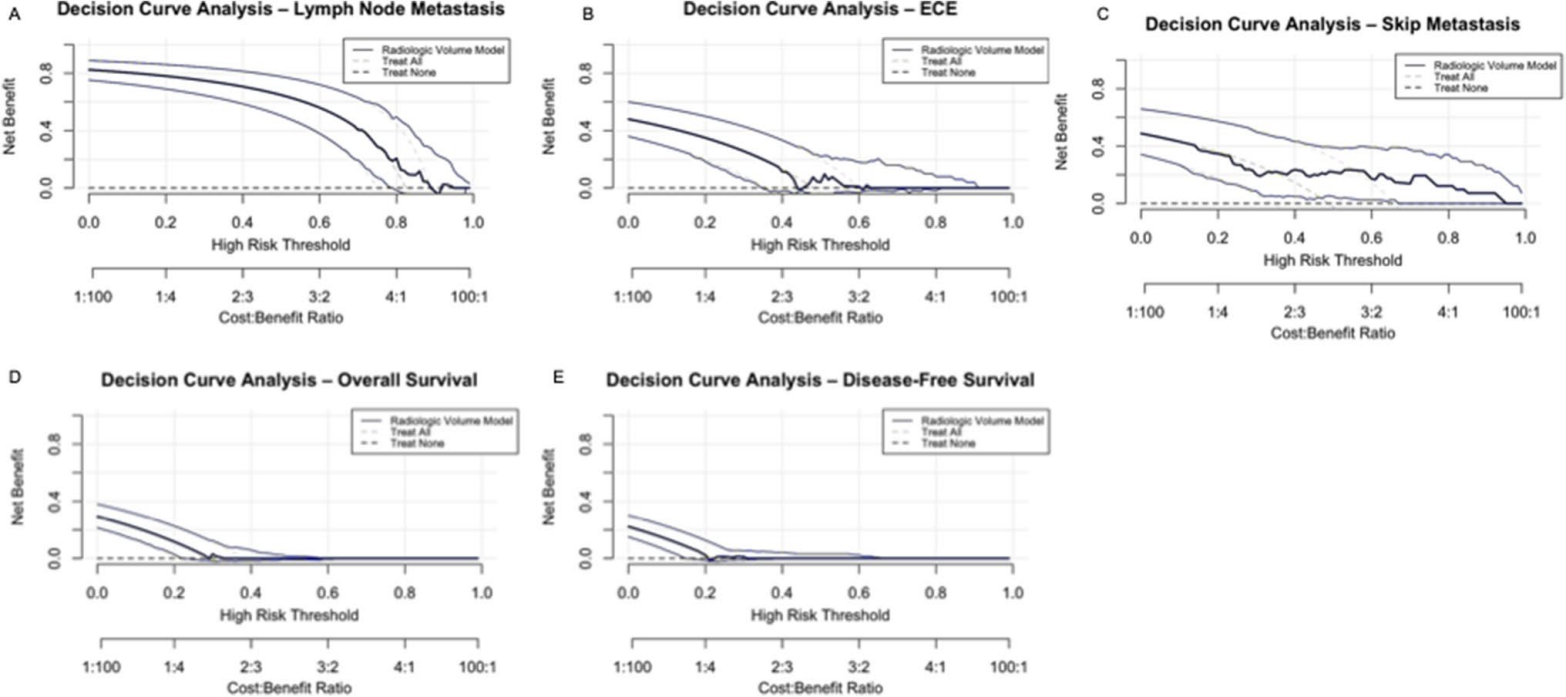
Decision-Curve Analysis for CT-Derived rTV in OSCC. Net clinical benefit curves compare a volume-based model with the default strategies of treating all or no patients across threshold probabilities for (**A**) lymph-node metastasis, (**B**) extranodal extension, and (**C**) skip metastasis. The volume model provides the greatest benefit at low-to-intermediate thresholds. CT indicates computed tomography; OSCC, oral squamous cell carcinoma

Decision-curve analysis demonstrated a consistent net clinical benefit of the rTV-based model across a range of clinically relevant threshold probabilities for LNM, compared with treat-all and treat-none strategies (Fig. 3A). For ENE, rTV provided modest net benefit at lower threshold probabilities, while no meaningful benefit was observed at higher thresholds (Fig. 3B).

### Non-linear association between radiologic tumor volume and skip metastasis

Patients with skip metastases had significantly higher log-transformed rTV than patients without skip metastases (*P* = 0.011). A logistic regression model incorporating a squared log-rTV term revealed a significant non-linear (U-shaped) association (*P* = 0.013), indicating increased probability of skip metastasis at both low and high tumor volumes. ROC analysis showed moderate discriminatory performance for rTV in predicting skip metastasis (AUC = 0.733). Decision-curve analysis suggested a potential net clinical benefit of rTV-based risk stratification for skip metastasis at low-to-intermediate risk thresholds (Fig. 3C).

### Exploratory analysis in cN0 patients

In cN0 patients, rTV was not significantly associated with occult LNM in univariable or multivariable exploratory analyses. These findings likely reflect limited statistical power and are therefore reported as hypothesis-generating.

### Agreeement between rTV and pTV

rTV and pTV showed poor agreement. Linear regression revealed no significant correlation (R^2^ = 0.0002), and Bland–Altman analysis demonstrated wide limits of agreement, particularly at higher tumor volumes. Despite this limited LNM and ENE (Fig. 3A–B), indicating complementary rather than interchangeable information.

### Exploratory survival analysis

**Fig. 4:**
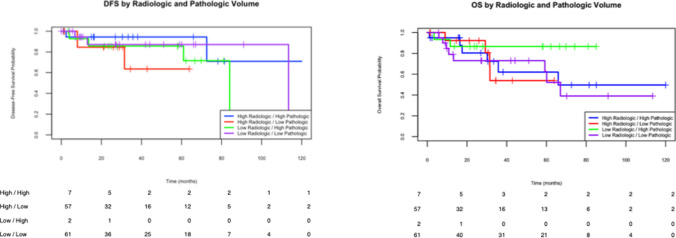
Survival According to Combined Radiologic and Pathologic Tumor Volume. Kaplan–Meier curves show disease-free survival (**A**) and overall survival (**B**) for four groups defined by median splits of CT-derived and pathologic volume: high/high, high/low, low/high, and low/low. Numbers at risk are provided beneath each plot. Survival did not differ significantly among groups (log-rank P = 0.43 for both outcomes), although the high-radiologic/low-pathologic group had the poorest numeric outcome, implying that CT volumetry may detect aggressive biology not captured histologically. CT indicates computed tomography; DFS, disease-free survival; OS, overall survival

Kaplan–Meier analyses showed no statistically significant differences in OS or DFS across combined rTV and pTV groups (*P* = 0.43 for both). However, patients with high rTV but low pTV exhibited numerically worse survival outcomes. Cox regression using continuous rTV demonstrated non-significant trends toward poorer OS and DFS. Decision-curve analyses for OS and DFS showed no meaningful net benefit across most threshold probabilities (Fig. 3D–E). Survival analyses were exploratory and conducted in the entire study cohort (Fig. 4).

## Discussion

This study underscores the clinical value of preoperative 3D tumor volumetry as a complementary tool in the risk stratification of OSCC. Given that the vast majority of tumors in our cohort arose from the tongue and floor of the mouth, the present findings primarily reflect volumetric behavior in these subsites. Traditional staging methods—such as maximum diameter and DOI —are often limited in capturing the true tumor burden. In contrast, radiologically derived tumor volume, obtained through CT segmentation, demonstrated robust independent predictive value. In our multivariable analysis, log-transformed tumor volume was significantly associated with LNM, and retained its significance even when adjusted for DOI and other confounders.

Beyond this, a notable non-linear association between volumetric tumor burden and skip metastasis risk was observed. A squared log-volume term revealed a U-shaped distribution, indicating increased likelihood of skip metastasis both in small and large tumors. These findings suggest distinct biological behaviors in low-volume tumor (possibly due to early, aggressive dissemination) and in bulky tumors (likely reflecting altered lymphatic drainage). Emerging evidence supports this interpretation: tumor budding has been associated with LNM and reduced DFS in early stage OSCC [13, 15]. Additionally, the detection of circulating and disseminated tumor cells indicates early systemic spread and portends poor prognosis [16]. In contrast, large tumor volumes might disrupt normal lymphatic architecture or promote alternative metastatic routes through stromal remodeling [14–16]. Together, these mechanisms may contribute to the atypical metastatic patterns observed at both ends of the tumor volume spectrum. Although tumor volume alone did not reach statistical significance in predicting occult lymph node metastasis in our cN0 cohort, volumetric assessment may still hold clinical relevance. DCA demonstrated a net clinical benefit for volume-based stratification across a plausible range of threshold probabilities, suggesting potential utility as a complementary parameter in nodal risk assessment. Notably, this benefit was most pronounced in the low-to-intermediate probability range, where clinical equipoise often exists regarding the choice between SLNB and END [23, 24]. Although volumetry did not independently predict occult nodal disease in cN0 patients, our findings suggest that volumetric information may enhance decision-making when integrated with existing clinical parameters. Patients with low tumor volume may derive limited benefit from END, and SLNB could represent a less invasive yet oncological sound alternative. This is consistent with prior studies demonstrating that SLNB achieves high sensitivity and specify in early-stage OSCC, with a favorable morbidity profile compared to END [17, 18]. Our data support a risk-adapted approach in which radiologic tumor volume serves as a stratification tool to guide nodal management in cN0 OSCC. Volumetry could serve as an adjunctive tool to guide nodal management in such ambiguous cases.

Interestingly, we observed limited agreement between radiologic and pathologic tumor volume measurements. Given that pathological volume was calculated after formalin fixation, tissue shrinkage likely contributed to the observed discrepancy [19, 20]. Formalin-induced dimensional changes have been widely reported, particularly affecting soft tissue architecture and leading to an underestimation of actual tumor size in histopathologic evaluation. Moreover, the process of sectioning and reconstructing tumor volume from two-dimensional slices introduces additional error, particularly in large or irregularly shaped tumors [21].In contrast, radiologic volumetry, especially when based on contrast-enhanced CT datasets, may overestimate viable tumor extent due to the inclusion of peritumoral inflammation, necrosis, or reactive tissue changes that are not tumor-specific [22, 23]. To our knowledge, such effects have been systematically investigated primarily in brain and abdominal tumors, but not yet in OSCC, highlighting a current gap in volumetric imaging validation for head and neck cancers. Collectively, these factors may explain the poor concordance between modalities and highlight the inherent methodological differences between radiologic and pathologic assessment. Despite this, radiologic volume outperformed pathologic estimates in predicting key clinical endpoints: AUC for LNM was higher, and it showed stronger discriminatory capacity for ENE and skip metastasis. While survival prediction using either modality remained modest volumetric imaging appeared to capture dimensions of tumor biology not fully reflected in histology.

Kaplan–Meier survival curves, though not statistically significant, highlighted clinically important trends. Patients with high radiologic but low pathologic volumes experienced worse outcomes, possibly reflecting metabolically active and invasive tumors not adequately sampled pathologically. Conversely, patients with low radiologic but high pathologic volumes had more favorable prognoses, perhaps reflecting more indolent disease components. These findings support the complementary roles of imaging and histology in characterizing tumor behavior.

DCA further substantiates the clinical utility of volumetry. The radiologic volume-based model demonstrated consistent net benefit across a wide range of threshold probabilities, particularly in the low-to-intermediate range (10–30%). This range corresponds to risk-averse clinical behavior—where physicians may choose intervention even at moderate estimated risk [24]. Thus, the added net benefit of volumetric modeling is especially valuable in early decision-making for cN0 patients, potentially improving the precision of nodal treatment recommendations. Integrating volumetric data into SLNB triaging could help identify patients who may benefit from more extensive dissection while sparing others from overtreatment.

Taken together, our results support the adoption of preoperative tumor volumetry as an adjunct in clinical staging, especially for tailoring nodal management. While conventional metrics like DOI and T stage remain foundational, volumetry offers an independent, quantifiable dimension that reflects tumor complexity and metastatic potential. Future incorporation into risk-adapted treatment algorithms—potentially in combination with artificial intelligence–driven segmentation and radiomics—could enhance patient stratification and individualize therapy.

A number of limitations require consideration. The retrospective, single-institution design and relatively small cohort, with few outcome events, limit statistical precision. The presence of variability in CT quality, in addition to the exclusion of suboptimal scans, could potentially result in the occurrence of selection bias. Another limitation is the limited concordance between radiologic and pathologic tumor volumes, likely influenced by fixation-related shrinkage and reconstruction methods. Although this was not the main focus of the present work, closer collaboration with pathology and systematic evaluation of shrinkage effects should be pursued in future prospective studies. Despite this, the volumetric associations exhibited both internal consistency and concordance with the findings of contemporary quantitative imaging studies in head and neck cancer, thereby suggesting the existence of a reproducible biological signal. This signal now requires confirmation in larger, multicentre cohorts. In conclusion, preoperative 3D volumetry represents a promising, clinically actionable imaging biomarker in OSCC, particularly in floor of mouth and tongue. It enriches current staging paradigms, augments nodal risk assessment, and holds potential to inform both surgical and non-surgical decision-making. As volumetric analysis becomes more accessible through evolving imaging platforms and software integration, its adoption in routine oncologic workflows should be increasingly considered, particularly in the nuanced care of cN0 patients, where its impact may be most profound.

## Supplementary Information

Below is the link to the electronic supplementary material.

Supplementary file1 (DOCX 19 KB)

## Data Availability

No datasets were generated or analysed during the current study.
