# Supplementary material for: Radiologic 3D tumor volume predicts cervical metastasis in oral squamous cell carcinoma
Source: Clin Oral Investig. 2026 Jul 23;30(8):357. doi: 10.1007/s00784-026-07043-y (PMC13395950; doi:10.1007/s00784-026-07043-y)
Supplement: Supplementary file 1 — Supplementary file1 (DOCX 19 KB) [file 784_2026_7043_MOESM1_ESM.docx]

**Supplementary Material**

**Radiologic 3D Tumor Volume as a Predictor of Lymph Node Metastasis in Oral Squamous Cell Carcinoma**

A.Schmitz^1^ ,V. Corneo^1^, C. Rendenbach^1^, C. Doll^1^, F. Elsholtz^2^, K. Kreutzer^1^, F. Mrosk^1^, M. Alfertshofer^1^, M. Heiland^1^, S. Koerdt^1^

1. Charité – Universitätsmedizin Berlin, corporate member of Freie Universität Berlin and Humboldt-Universität zu Berlin, Department of Oral and Maxillofacial Surgery, Augustenburger Platz 1, 13353 Berlin, Germany

Corresponding author: Alina Marie Schmitz

Alina-marie.schmitz@charite.de

**Supplementary Material**

Supplementary Table 1: Multivariable Logistic Regression Predicting Pathologic Lymph-Node Metastasis

| Predictor | OR (95 % CI) | *z* | *P* |
| --- | --- | --- | --- |
| Radiologic tumor volume (log cm³) | 3.00 (1.21 – 7.68) | 2.41 | *.02* * |
| Depth of invasion, mm | 1.04 (0.95 – 1.13) | 0.82 | .41 |
| Grade 2 vs *Grade 1* | 2.38 (0.47 – 12.04) | 1.03 | .30 |
| Grade 3 vs *Grade 1* | 0.75 (0.08 – 7.23) | –0.25 | .80 |
| Floor of mouth vs *Tongue* | 1.66 (0.52 – 5.30) | 0.86 | .42 |
| Other subsites† vs *Tongue* | 1.45 (0.28 – 7.59) | 0.44 | .66 |
| Age, per year | 1.02 (0.97 – 1.07) | 0.62 | .53 |

Table 1: C-statistic = 0.78; Hosmer–Lemeshow P = .72. *P < .05. †“ Other subsites” combines upper/lower gingiva. Abbreviations: CI, confidence interval; OR, odds ratio.
